# Investigating gender disparities in urological care for non‐muscle invasive bladder cancer (NMIBC)

**DOI:** 10.1002/bco2.70245

**Published:** 2026-08-03

**Authors:** Aleyna Aydin, Amin Salaah Abdulmajeed, Fatima Betul Bekiroglu, Mehmet Kocak, Pilar Laguna, Rahim Horuz, Selami Albayrak, Valentin Pavlov, Guohua Zeng, Jean de la Rosette, the global NMIBC survey collaboration

**Affiliations:** ^1^ School of Medicine Istanbul Medipol University Istanbul Türkiye; ^2^ International School of Medicine Istanbul Medipol University Istanbul Türkiye; ^3^ Multi‐Omics Design and Analysis Studio (MODAS‐SABITA) Istanbul Medipol University Istanbul Türkiye; ^4^ Department of Urology Medipol Mega University Hospital Istanbul Türkiye; ^5^ Bashkir State Medical University Ufa Russia; ^6^ Department of Urology and Guangdong Key Laboratory of Urology The First Affiliated Hospital of Guangzhou Medical University Guangzhou China

**Keywords:** diagnostics, gender disparity, guidelines, NMIBC, non‐muscle invasive bladder cancer, survey, treatment, TURBT

## Abstract

**Objective:**

This study aims to explore possible gender disparities in urological diagnostic and therapeutic care for NMIBC, particularly in the use of enhanced endoscopic imaging techniques.

**Materials and Methods:**

An international survey in seven languages was distributed to all members of the Société Internationale d'Urologie (SIU). We captured specifications related to TURBT performance, attitudes towards the use of enhanced imaging and adherence to international guidelines for non‐muscle invasive bladder cancer. Data were analysed using descriptive statistics. Gender disparities were assessed using propensity score matching (PSM) for age, working environment, subspecialisation and years of experience.

**Results:**

A total of 3595 SIU members from 91 countries participated (response rate 35%). Male participants performed a higher volume of TURBTs per month (*p* = 0.0087). In accordance with guidelines, taking biopsies from normal or abnormal appearing tissue was rated equally among males and females (*p* = 0.68 for each). No significant gender‐based differences were observed in attitudes towards the benefits of enhanced imaging techniques, including PPD, NBI and Image 1S. This was consistent for identification of satellite lesions (*p* = 0.99) and completeness of TURBT, including resection of margins (*p* = 0.07) and tumour floor (*p* = 0.66). Guidelines were followed equally by both sexes, with a strong preference for EAU guidelines. Similar findings were observed among residents and fellows.

**Conclusion:**

No significant gender‐related disparities were found in urological care for NMIBC, including the use of enhanced imaging techniques. These findings apply to both urologists and trainees, with comparable adherence to guidelines.

## 1 INTRODUCTION

White light (WL) transurethral resection of bladder tumour (TURBT) is the standard modality of care for non‐muscle invasive bladder cancer (NMIBC). However, utilizing only white light as the main imaging technique, lesions can often be missed due to their circumspect nature. Recurrence rate following WL TURBT is significant and may be a result of unintentional incomplete resection.^1^, ^2^ Improving the quality of TURBT is essential to achieving persistent results and lowering recurrence rates, which depend greatly on the individual operating urologists' experience with TURBT.^3^ Performing and accomplishing such a procedure requires highly competent specialists with sufficient training and expertise to ensure a complete resection of all visible tumours and suspicious areas. The undertaking of TURBT constitutes a procedure of notable intricacy, demanding a nuanced approach and a thoughtful utilization of resources. Performed by specialized urologists, this process encompasses a set of challenges that necessitate a comprehensive understanding and an exceptional application of skills. In the pursuit of improving TURBT, a multifaceted approach is essential to enhance detection precision and treatment optimization. First and foremost, the integration of advanced technology, such as the utilization of instruments and new energy sources, alongside modifications in TURBT techniques and the enhanced endoscopic imaging capabilities such as Image 1S, Photodynamic diagnosis (PPD) and Narrow Band Imaging (NBI), proves instrumental.^4^ Furthermore, the strides made in histopathological evaluation contribute significantly to diagnostic accuracy and individual treatment strategies of NMIBC. Another crucial aspect involves quality control, emphasizing the importance of awareness and specialized training to ensure consistently high standards in the application of these advancements in urological care of NMIBC. Considering these techniques, numerous studies suggest differences in management preferences of NMIBC based on gender.^5^ A growing body of evidence indicates gender effects across medical fields, emphasizing the importance of considering gender‐specific variations, especially regarding the preferred treatment techniques that are employed by specialists.^6^

However, the relationship between gender, urological care for NMIBC and its potential influence on the preferred utilization of enhanced endoscopic imaging techniques when treating NMIBC has not been researched before. Our study aims to explore possible gender differences in urological care for NMIBC, utilizing enhanced imaging techniques by taking a close look at how individuals of each gender adhere to established guidelines, the number of TURBTs performed per month, the potential benefits of each imaging technique and so forth, considering both residents and specialists of each gender.

## 2 MATERIALS AND METHODS

A cross‐sectional online survey was sent out to members of the Société Internationale d'Urologie (SIU), spanning data collection between May and July 2022. Adherence to the Consensus‐Based Checklist for Reporting of Survey Studies (CROSS) guided the formulation of survey questions, ensuring both replicability and comprehensibility. To preserve linguistic accuracy, native bilingual urology specialists proficient in English, Chinese, French, Portuguese, Russian, Spanish and Turkish translated the survey. Participation was voluntary, facilitated through region‐specific, single‐access links embedded in emails sent to SIU members. Since the data were collected anonymised, no IRB approval was requested.

The survey specifically targeted healthcare professionals possessing the qualifications to either actively engage in or oversee procedures employing the discussed imaging methods. Utilizing the SurveyMonkey platform, a robust tool provided by Momentive Inc. (accessible at www.momentive.ai), the survey successfully navigated the global landscape, capturing insights from a diverse array of urology professionals.

Designed with data integrity in mind, the survey structure mandated completion of all questions before submission, preventing potential imbalances. The questionnaire featured 22 diverse questions, incorporating multi‐select matrices, Likert scales, sliders and multiple‐choice queries, with three providing an ‘other’ option. For gender‐based analysis compatibility, a propensity score matching (PSM) approach was utilized to match each female urologist with as many male urologists as possible (1:many matches) based on age (calliper width of 0.25), and exact matched on practice setting, subspecialisation and years of experience, using SAS PSMATCH procedure. This matching strategy was conducted independently for residents and fellows. This matching strategy resulted in 148 female urologists with 946 male counterparts and 73 female residents/fellows with 334 male counterparts, facilitating a more nuanced examination of gender‐related dynamics within the dataset. This statistical methodology ensured a more equitable comparison between the groups of interest because of male–female matching.

Subsequent data analysis presented findings through descriptive statistics, providing nuanced insights into this diverse and globally representative urological cohort. The tables were generated using row‐ or column‐percentages whichever makes more sense in the presentation. To assess the differential characteristic of practice between males and females, Chi‐Square was used. Type‐1 error rate of 0.05 was considered as statistically significant without multiplicity adjustment; therefore, the results must be considered within the hypothesis generating context. For all analysis, SAS® Version 9.4 (Cary, North Carolina, USA) was used.

## 3 RESULTS

A total of 3595 questionnaires were received from 91 countries (35% response rate), constituting a richly diverse dataset with Asia contributing 45.9% and Europe 22.9% of the overall responses (Table 1). Within this globally inclusive survey, a pronounced male majority (~92%) and a predominant professional affiliation with urology (96.7%) characterized the participating cohort. The average age of participants converged at 46.2 ± 12.2 years. Distribution of the responses of participants to clinical practice questions such as TURBT frequency is presented in Table S1. After propensity score matching, our analysis cohort is composed of 1094 participants, with an average age of 42.6 ± 9.0 versus 43.5 ± 8.9 for females and males respectively with no age difference between males and females (*p* = 0.17) and other matching characteristics also well‐balanced (Table 2). In the matched residents and fellow's cohort, we had 73 females and 334 males with average ages of 30.2 ± 3.3 versus 30.4 ± 2.9 for females and males respectively with no age difference between males and females (*p* = 0.34).

**TABLE 1:** All participants distributed by gender versus continent, years of clinical practice, stage of career and practice setting.

	All participants		Female		Male		Prefer not to answer	
	*N*	Col %	*N*	Col %	*N*	Col %	*N*	Col %
All participants	3595	100.0	279	100.0	3304	100.0	12	100.0
Continent								
1: North America	158	4.4	18	6.5	140	4.2	.	.
2: Latin America	624	17.4	57	20.4	567	17.2	.	.
3: Europe	825	22.9	100	35.8	719	21.8	6	50.0
4: Africa	337	9.4	7	2.5	329	10.0	1	8.3
5: Asia	1651	45.9	97	34.8	1549	46.9	5	41.7
How many years have you been in the clinical practice?								
0–5 years	609	16.9	94	33.7	512	15.5	3	25.0
6–10 years	594	16.5	52	18.6	542	16.4	.	.
11–15 years	527	14.7	52	18.6	474	14.3	1	8.3
16–20 years	459	12.8	34	12.2	421	12.7	4	33.3
>20 years	1406	39.1	47	16.8	1355	41.0	4	33.3
Which one of the following best describes the stage of your career?								
Other	94	2.6	20	7.2	74	2.2	.	.
I am a qualified (general) urologist	2050	57.0	136	48.7	1910	57.8	4	33.3
I am a qualified uro‐oncologist	610	17.0	24	8.6	584	17.7	2	16.7
I am a qualified endourologist	304	8.5	9	3.2	293	8.9	2	16.7
I am a resident in training	445	12.4	75	26.9	368	11.1	2	16.7
I am in a urology fellowship programme	68	1.9	11	3.9	56	1.7	1	8.3
Unknown	24	0.7	4	1.4	19	0.6	1	8.3
Which one of the following best describes your practice setting?								
.	338	9.4	39	14.0	296	9.0	3	25.0
Academic/university hospital	1530	42.6	130	46.6	1393	42.2	7	58.3
Public non‐academic hospital	756	21.0	63	22.6	691	20.9	2	16.7
Private practice (office and/or hospital)	971	27.0	47	16.8	924	28.0	.	.

**TABLE 2:** Matched‐cohort between gender including continent, years of clinical practice, stage of career and practice setting.

	All participants		Female		Male		Chi‐square *p*‐value
	*N*	Col %	*N*	Col %	*N*	Col %	
All participants	1094	100.0	148	100.0	946	100.0	
Continent							
1: North America	28	2.6	9	6.1	19	2.0	
2: Latin America	145	13.3	26	17.6	119	12.6	
3: Europe	249	22.8	52	35.1	197	20.8	
4: Africa	117	10.7	4	2.7	113	11.9	
5: Asia	555	50.7	57	38.5	498	52.6	
How many years have you been in the clinical practice? (q4_years_in_practice)							
0–5 years	153	14.0	26	17.6	127	13.4	0.64
6–10 years	226	20.7	31	20.9	195	20.6	
11–15 years	230	21.0	31	20.9	199	21.0	
16–20 years	200	18.3	27	18.2	173	18.3	
>20 years	285	26.1	33	22.3	252	26.6	
Which one of the following best describes the stage of your career? (q5_career_stage)							
I am a qualified (general) urologist	883	80.7	118	79.7	765	80.9	0.81
I am a qualified uro‐oncologist	163	14.9	22	14.9	141	14.9	
I am a qualified endourologist	48	4.4	8	5.4	40	4.2	
Which one of the following best describes your practice setting? (q6_practice_setting)							
Academic/university hospital	503	46.0	66	44.6	437	46.2	0.73
Public non‐academic hospital	296	27.1	44	29.7	252	26.6	
Private practice (office and/or hospital)	295	27.0	38	25.7	257	27.2	

Male participants exhibit a notably higher volume of transurethral resection of bladder tumours (TURBTs) per month, with a statistically significant *p*‐value of 0.0087. In adherence to established guidelines, the act of taking biopsies, irrespective of whether from normal or abnormal‐looking tissue, garnered equivalent ratings for both males and females, with *p*‐values of 0.68 each (Table 3).

**TABLE 3:** Gender‐specific comparisons related to TURBT including the use of enhanced imaging.

	Female		Male		Chi‐square *p*‐value
	*N*	Row %	*N*	Row %	
How many TURBT for bladder cancer (primary or secondary) do you perform per month?					
None	10	9.7	35	5.5	0.0087
1–2	30	29.1	186	29.2	
3–5	31	30.1	223	35.1	
6–10	18	17.5	94	14.8	
>10	14	13.6	98	15.4	
I usually take biopsies only from abnormal looking urothelium.					
Agree	71	68.9	425	66.8	0.68
Disagree	32	31.1	211	33.2	
I take mapping biopsies in >50% of cases with white light from normal looking urothelium.					
Agree	39	37.9	244	38.4	0.68
Disagree	64	62.1	392	61.6	
I only take mapping biopsies from normal looking urothelium with white light if cytology or urinary marker test is positive.					
Agree	64	62.1	441	69.3	0.056
Disagree	39	37.9	195	30.7	
I almost never take bladder biopsies.					
Agree	18	17.5	70	11.0	0.074
Disagree	85	82.5	566	89.0	
There is no real benefit.					
Agree	11	10.7	102	16.0	0.83
Disagree	63	61.2	388	61.0	
I do not know	29	28.2	146	23.0	
To identify satellite lesions for a lower recurrence rate.					
Agree	76	73.8	493	77.5	0.79
Disagree	7	6.8	33	5.2	
I do not know	20	19.4	110	17.3	
To perform more complete resections in the outer tumour margin.					
Agree	69	67.0	461	72.5	0.70
Disagree	14	13.6	70	11.0	
I do not know	20	19.4	105	16.5	
To perform more complete resection on the tumour floor.					
Agree	52	50.5	347	54.6	0.66
Disagree	25	24.3	157	24.7	
I do not know	26	25.2	132	20.8	
There is a benefit when using PDD.					
Agree	46	44.7	283	44.5	0.62
Disagree	11	10.7	65	10.2	
I do not know	46	44.7	288	45.3	
There is a benefit when using NBI.					
Agree	46	44.7	333	52.4	0.16
Disagree	8	7.8	57	9.0	
I do not know	49	47.6	246	38.7	
There is a benefit when using IMAGE 1S.					
Agree	23	22.3	229	36.0	0.028
Disagree	8	7.8	52	8.2	
I do not know	72	69.9	355	55.8	
There is no benefit for PDD or NBI or IMAGE 1S.					
Agree	9	8.7	89	14.0	0.16
Disagree	42	40.8	283	44.5	
I do not know	52	50.5	264	41.5	
Multiple bladder tumours.					
Agree	72	69.9	431	67.8	0.92
Disagree	12	11.7	81	12.7	
I do not know	19	18.4	124	19.5	
Larger bladder tumour including smaller satellite lesions.					
Agree	71	68.9	435	68.4	0.65
Disagree	10	9.7	83	13.1	
I do not know	22	21.4	118	18.6	
In patients with pTa tumours to reduce recurrence rate.					
Agree	51	49.5	367	57.7	0.28
Disagree	24	23.3	132	20.8	
I do not know	28	27.2	137	21.5	
In patients with pT1 tumours to reduce recurrence rate.					
Agree	65	63.1	425	66.8	0.78
Disagree	16	15.5	77	12.1	
I do not know	22	21.4	134	21.1	
In patients with CIS to better diagnose and treat CIS.					
Agree	90	87.4	529	83.2	0.39
Disagree	.	.	22	3.5	
I do not know	13	12.6	85	13.4	
Which guidelines do you predominantly follow for the diagnosis and treatment of NMIBC? (Please select the one that best applies)					
The national guidelines prepared by my own society.	15	14.6	84	13.2	0.31
The national guidelines modified from the AUA, the EAU, the NCCN or similar guidelines.	30	29.1	205	32.2	
The AUA guidelines	6	5.8	28	4.4	
The EAU guidelines	43	41.7	267	42.0	
The NCCN guidelines	4	3.9	26	4.1	
I do not follow specific guidelines.	5	4.9	26	4.1	

Examining attitudes towards the benefits of enhanced imaging techniques, such as PPD, NBI and Image 1S, no statistically significant gender‐based differences emerged from the data analysis. This lack of distinction is particularly evident concerning the identification of satellite lesions (*p* = 0.99) and the comprehensive nature of TURBT, encompassing resection of margins (*p* = 0.070) and the tumour floor (*p* = 0.66). Furthermore, the commitment to adhering to guidelines stands out as a unifying factor between both male and female participants, with a marked inclination towards following the European Association of Urology (EAU) guidelines. These findings collectively underscore our finding that the utilization of enhanced imaging techniques does not vary according to the gender of the practicing specialist (Table 3).

Results for residents and fellows are presented in Table S2 and are generally in line with the above‐mentioned results for the more senior urologists.

## 4 DISCUSSION

No substantial gender‐associated disparity in urological care was found in either using enhanced endoscopic imaging techniques or following (inter)national guidelines in diagnosing and treatment of NMIBC. This applies to urologists as well as residents.

Gender‐based differences in medical practice and decision‐making have been extensively documented across various specialties, with female physicians often demonstrating distinct communication styles, clinical priorities and procedural preferences compared to their male counterparts. For instance, female physicians are more likely to allocate extended time to patient consultations, prioritize preventive care and adhere rigorously to evidence‐based guidelines.^7^, ^8^ These tendencies align with broader findings that female clinicians exhibit greater diagnostic caution, lower tolerance for clinical uncertainty and a higher propensity for ordering diagnostic tests.^9^ Such patterns reflect a patient‐centred approach that emphasizes shared decision‐making and holistic care—a framework that may inherently shape diagnostic and therapeutic choices. Interestingly, the current survey did not demonstrate any significant differences between genders for either urologists or residents in training. The question may arise if this is the case in urological care or specifically for diagnosis and/or treatment of NMIBC.

In urology, these gender‐driven differences extend beyond primary care into subspecialization and procedural focus. Female urologists are more likely to pursue functional urology and female pelvic medicine, whereas male urologists dominate oncological and complex reconstructive procedures.^10^, ^11^ This divergence is influenced by a combination of physician preferences, patient‐driven gender biases and systemic barriers such as unequal access to mentorship and leadership roles.^12^, ^13^ For example, male urologists historically dominated prostate cancer surgery, partly due to patient perceptions of technical expertise, while female urologists have carved niches in pelvic organ prolapse and incontinence management.^10^, ^11^

**FIGURE 1:**
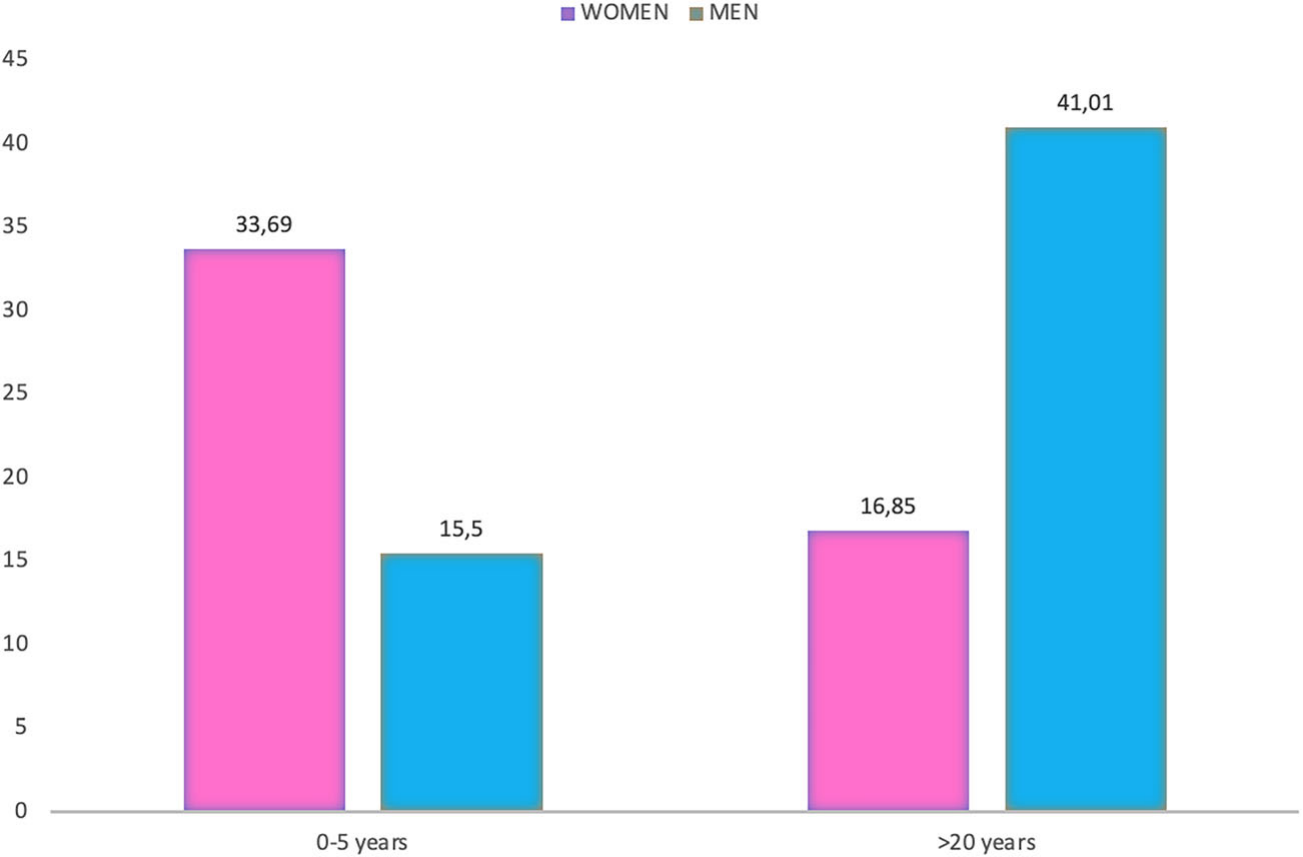
Gender distribution across career stages in urology; distribution of male and female urologists stratified by years of clinical practice (0–5 years vs. >20 years), illustrating the higher proportion of women in earlier career stages and the predominance of men among senior practitioners.

Nevertheless, it is important to acknowledge that gender‐based trends in male‐dominated specialties such as urology are undergoing rapid change. Our survey reflects this shift, showing that the majority of residents and a significant proportion of fellows currently in urological training are female (Figure 1). This generational transition raises the question of whether emerging female urologists will differ from their predominantly male predecessors in terms of risk perception and clinical decision‐making. The notable increase in female representation within training programs may signal a broader evolution in practice patterns. Alternatively, the discrepancy between earlier findings and the present results may suggest that factors beyond gender—such as evolving institutional standards, guideline‐driven practice or technological integration—are mitigating previously observed differences.

The assumption that gender invariably dictates practice patterns is increasingly challenged in contexts where institutional protocols, technological advancements and standardized training mitigate individual variability. For instance, in radiology—a field governed by strict imaging guidelines—studies reveal minimal gender‐based differences in the utilization of advanced modalities like MRI or CT scans.^14^ These findings highlight the role of environmental factors in overriding individual tendencies, particularly in specialties where evidence‐based frameworks dominate.

It is believed that female physicians' specialties tend to adopt more conservative approaches when faced with clinical ambiguity, such as ordering additional imaging studies or delaying invasive interventions.^6^, ^15^ In surgical settings, female surgeons have been reported to prioritize thorough intraoperative assessments and visualization before proceeding with complex manoeuvres.^16^ These patterns suggest that gender may influence not only what procedures are performed but also how clinical decisions are cognitively framed. In the present work, female urologists appear to make the same decision in using enhanced imaging for visualization and position themselves equally towards their male counterparts in taking biopsies and/or resecting NMIBCs.

These results align with emerging evidence that gender differences in technology adoption diminish when innovations are perceived as patient‐centric rather than discretionary. Enhanced imaging, which enhances diagnostic accuracy and reduces unnecessary invasive procedures, may similarly align with patient‐centred priorities shared across genders. This suggests that when technologies are framed as tools to optimize care—rather than as discretionary ‘add‐ons’—gender‐based variations in utilization may fade.

Another important factor may be the foundation laid in building strong Evidence Based Guidelines. In urology, the integration of enhanced imaging technologies—such as multiparametric MRI (mpMRI) for prostate cancer or dynamic ultrasonography for pelvic floor dysfunction—has been guided by rapidly evolving, protocol‐driven guidelines. Recent studies demonstrated that adherence to prostate imaging reporting and data system (PI‐RADS) standards among urologists showed no gender‐based variation, as both male and female providers prioritized guideline concordance over subjective risk perception. This mirrors findings in primary care, where the implementation of electronic feedback systems—designed to promote adherence to evidence‐based guidelines—eliminated gender disparities in physician decision‐making for patients presenting with identical symptoms, suggesting that standardization through technology can reduce within‐physician biases.^17^ Such examples suggest that in technology‐driven domains, gender differences may dissipate when clinical decisions are anchored to objective criteria. This is confirmed in the present work, which shows that both genders follow the guidelines equally.

Structural barriers, such as unequal access to mentorship and leadership, have historically perpetuated gender disparities in procedural confidence and technology adoption. Female urologists, particularly in male‐dominated environments, report fewer opportunities to lead departments overseeing equipment procurement or training programmes.^18^ However, generational shifts in medical training are gradually eroding these barriers. Recent efforts to standardize residency curricula and expand access to simulation‐based training have contributed to a more equitable educational environment, offering all trainees—regardless of gender—greater exposure to advanced imaging and procedural technologies. Nevertheless, earlier studies have documented persistent gaps in procedural confidence among female residents, often linked to limited hands‐on opportunities or lack of role models.^19^, ^20^, ^21^ Institutional efforts to promote equitable mentorship—such as structured peer‐mentoring networks and gender‐balanced leadership committees—are further narrowing these gaps.^22^, ^23^ Therefore, it comes as no surprise that we did not find any significant differences between male and female urology residents.

Patient preferences also play a nuanced role in shaping practice patterns. While historical studies noted patient biases towards gender‐concordant urologists for sensitive conditions like erectile dysfunction or pelvic prolapse,^24^, ^25^ contemporary data suggest declining gender‐based preferences in technology‐driven care.^26^, ^27^ Recent research supports this trend. A 2023 study of underserved urology patients in New York City found that the majority had no gender preference when selecting a urologist, emphasizing provider competence over concordance.^28^ Similarly, an observational study in telemedicine revealed minimal gender‐based differences in patient satisfaction or visit dynamics, suggesting that the virtual, technology‐driven nature of care may further diminish the salience of provider gender.^26^ This shift may reduce pressure on urologists to align their practice patterns with perceived gender norms, fostering more uniform utilization of innovations like enhanced imaging.

The absence of gender disparities in our study underscores the importance of contextualizing existing literature. While prior research emphasizes gender as a determinant of practice patterns, our findings suggest that in domains governed by strong evidence, standardized training and collaborative technologies, physician gender may exert minimal influence. This has critical implications for equity in technological adoption: If enhanced imaging utilization is gender‐neutral, its benefits may be more uniformly accessible to patients across providers.

Finally, institutional culture remains a critical moderating factor. Hospitals with robust mentorship programs, gender‐balanced leadership and transparent technology procurement processes may foster more equitable practice environments. Qualitative investigations into these cultures—particularly in academic versus community settings—could identify best practices for minimizing gender‐based variability.

The strength of the survey is a high response rate and includes a significant number of female urologists and residents in training. This allows for matching both genders at different levels in good conditions. But the present work has several inherent limitations. First, for obvious reasons, females were significantly younger than their male counterparts. Hence, the survey was not designed to go in depth on issues such as specific training and attitudes towards technologies in general. We strived to reduce these limitations by utilizing male–female matching on key characteristics. Another limitation of the survey was that participation from Latin America, North America and Africa was less than 15% each, which potentially limits the generalizability of our findings.

## 5 CONCLUSION

The historical narrative of gender‐driven differences in medical practice is both nuanced and context‐dependent. While female and male physicians have demonstrated divergent approaches in communication, procedural focus and risk tolerance, our study highlights that these differences do not universally extend to enhanced imaging utilization in urology. In protocol‐driven, technology‐intensive domains, adherence to evidence‐based guidelines and collaborative decision‐making frameworks may override individual gender‐related tendencies. This finding challenges assumptions about gender's role in clinical practice and underscores the transformative potential of standardized training, equitable mentorship and patient‐centred technological integration. As the medical workforce becomes increasingly gender‐balanced, continued focus on systemic equity—rather than individual differences—will ensure that technological advancements benefit all patients equitably.

## AUTHOR CONTRIBUTIONS

Aleyna Aydin, Amin Salaah Abdulmajeed, Fatima Betul Bekiroglu, Rahim Horuz, Selami Albayrak, Valentin Pavlov and Guohua Zeng contributed to writing and revising the manuscript. Mehmet Kocak contributed to the study design, manuscript writing and supervision. Pilar Laguna contributed to manuscript writing, revision and supervision. Jean de la Rosette contributed to study design, manuscript writing and supervision. All authors reviewed and approved the final manuscript.

## CONFLICT OF INTEREST STATEMENT

Aleyna Aydin, Amin Salaah Abdulmajeed, Fatima Betul Bekiroglu, Mehmet Kocak, Pilar Laguna, Rahim Horuz, Selami Albayrak, Valentin Pavlov, Guohua Zeng and Jean de la Rosette declare that they have no financial or personal relationships that could have appeared to influence the work reported in this paper.

## Supporting information


**Table S1.** All cohort with their responses to clinical practice questions.
**Table S2:** Gender‐specific comparisons related to TURBT including the use of enhanced imaging in the Residents/Fellows cohort.

## Data Availability

The data underlying this article were collected through an anonymized international survey distributed to members of the Société Internationale d'Urologie (SIU). Due to privacy and confidentiality agreements with the SIU, the data are not publicly available. However, the dataset can be made available from the corresponding author upon reasonable request and with permission from the SIU.
