# Supplementary material for: Investigating gender disparities in urological care for non‐muscle invasive bladder cancer (NMIBC)
Source: BJUI Compass. 2026 Aug 3;7(8):e70245. doi: 10.1002/bco2.70245 (PMC13434217; doi:10.1002/bco2.70245)
Supplement: Supplementary file 1 [file BCO2-7-e70245-s001.docx]

**Table A1.** All cohort with their responses to clinical practice questions

|  | All Participants | | Female | | Male | | Prefer not to answer | |
| --- | --- | --- | --- | --- | --- | --- | --- | --- |
|  | N | Col % | N | Col % | N | Col % | N | Col % |
| All Participants | 3595 | 100.0 | 279 | 100.0 | 3304 | 100.0 | 12 | 100.0 |
| How many TURBT for bladder cancer (primary or secondary) do you perform per month? | 338 | 9.4 | 39 | 14.0 | 296 | 9.0 | 3 | 25.0 |
| . |  |  |  |  |  |  |  |  |
| None | 314 | 8.7 | 49 | 17.6 | 264 | 8.0 | 1 | 8.3 |
| 1-2 | 960 | 26.7 | 60 | 21.5 | 897 | 27.1 | 3 | 25.0 |
| 3-5 | 997 | 27.7 | 68 | 24.4 | 925 | 28.0 | 4 | 33.3 |
| 6-10 | 519 | 14.4 | 35 | 12.5 | 484 | 14.6 | . | . |
| >10 | 467 | 13.0 | 28 | 10.0 | 438 | 13.3 | 1 | 8.3 |
| I usually take biopsies only from abnormal looking urothelium. | 823 | 22.9 | 73 | 26.2 | 746 | 22.6 | 4 | 33.3 |
| . |  |  |  |  |  |  |  |  |
| Agree | 1931 | 53.7 | 127 | 45.5 | 1797 | 54.4 | 7 | 58.3 |
| Disagree | 841 | 23.4 | 79 | 28.3 | 761 | 23.0 | 1 | 8.3 |
| I take mapping biopsies in >50% of cases with White Light from normal looking urothelium. | 885 | 24.6 | 78 | 28.0 | 803 | 24.3 | 4 | 33.3 |
| . |  |  |  |  |  |  |  |  |
| Agree | 1139 | 31.7 | 85 | 30.5 | 1049 | 31.7 | 5 | 41.7 |
| Disagree | 1571 | 43.7 | 116 | 41.6 | 1452 | 43.9 | 3 | 25.0 |
| I only take mapping biopsies from normal looking urothelium with White Light if cytology or urinary marker test is positive. | 902 | 25.1 | 81 | 29.0 | 817 | 24.7 | 4 | 33.3 |
| . |  |  |  |  |  |  |  |  |
| Agree | 1822 | 50.7 | 126 | 45.2 | 1691 | 51.2 | 5 | 41.7 |
| Disagree | 871 | 24.2 | 72 | 25.8 | 796 | 24.1 | 3 | 25.0 |
| I almost never take bladder biopsies. | 968 | 26.9 | 78 | 28.0 | 885 | 26.8 | 5 | 41.7 |
| . |  |  |  |  |  |  |  |  |
| Agree | 395 | 11.0 | 42 | 15.1 | 350 | 10.6 | 3 | 25.0 |
| Disagree | 2232 | 62.1 | 159 | 57.0 | 2069 | 62.6 | 4 | 33.3 |
| There is no real benefit. | 1159 | 32.2 | 97 | 34.8 | 1058 | 32.0 | 4 | 33.3 |
| . |  |  |  |  |  |  |  |  |
| Agree | 396 | 11.0 | 24 | 8.6 | 370 | 11.2 | 2 | 16.7 |
| Disagree | 1464 | 40.7 | 112 | 40.1 | 1347 | 40.8 | 5 | 41.7 |
| I do not know | 576 | 16.0 | 46 | 16.5 | 529 | 16.0 | 1 | 8.3 |
| To identify satellite lesions for a lower recurrence rate. | 1109 | 30.8 | 93 | 33.3 | 1012 | 30.6 | 4 | 33.3 |
| . |  |  |  |  |  |  |  |  |
| Agree | 1910 | 53.1 | 144 | 51.6 | 1760 | 53.3 | 6 | 50.0 |
| Disagree | 164 | 4.6 | 10 | 3.6 | 153 | 4.6 | 1 | 8.3 |
| I do not know | 412 | 11.5 | 32 | 11.5 | 379 | 11.5 | 1 | 8.3 |
| To perform more complete resections in the outer tumor margin. | 1128 | 31.4 | 90 | 32.3 | 1034 | 31.3 | 4 | 33.3 |
| . |  |  |  |  |  |  |  |  |
| Agree | 1826 | 50.8 | 140 | 50.2 | 1680 | 50.8 | 6 | 50.0 |
| Disagree | 261 | 7.3 | 19 | 6.8 | 241 | 7.3 | 1 | 8.3 |
| I do not know | 380 | 10.6 | 30 | 10.8 | 349 | 10.6 | 1 | 8.3 |
| To perform more complete resection on the tumor floor. | 1141 | 31.7 | 92 | 33.0 | 1045 | 31.6 | 4 | 33.3 |
| . |  |  |  |  |  |  |  |  |
| Agree | 1364 | 37.9 | 105 | 37.6 | 1253 | 37.9 | 6 | 50.0 |
| Disagree | 587 | 16.3 | 39 | 14.0 | 547 | 16.6 | 1 | 8.3 |
| I do not know | 503 | 14.0 | 43 | 15.4 | 459 | 13.9 | 1 | 8.3 |
| There is a benefit when using PDD. | 1131 | 31.5 | 90 | 32.3 | 1037 | 31.4 | 4 | 33.3 |
| . |  |  |  |  |  |  |  |  |
| Agree | 1126 | 31.3 | 94 | 33.7 | 1028 | 31.1 | 4 | 33.3 |
| Disagree | 237 | 6.6 | 15 | 5.4 | 221 | 6.7 | 1 | 8.3 |
| I do not know | 1101 | 30.6 | 80 | 28.7 | 1018 | 30.8 | 3 | 25.0 |
| There is a benefit when using NBI. | 1140 | 31.7 | 93 | 33.3 | 1043 | 31.6 | 4 | 33.3 |
| . |  |  |  |  |  |  |  |  |
| Agree | 1275 | 35.5 | 89 | 31.9 | 1181 | 35.7 | 5 | 41.7 |
| Disagree | 226 | 6.3 | 11 | 3.9 | 214 | 6.5 | 1 | 8.3 |
| I do not know | 954 | 26.5 | 86 | 30.8 | 866 | 26.2 | 2 | 16.7 |
| There is a benefit when using IMAGE1S. | 1171 | 32.6 | 97 | 34.8 | 1070 | 32.4 | 4 | 33.3 |
| . |  |  |  |  |  |  |  |  |
| Agree | 869 | 24.2 | 51 | 18.3 | 814 | 24.6 | 4 | 33.3 |
| Disagree | 205 | 5.7 | 13 | 4.7 | 191 | 5.8 | 1 | 8.3 |
| I do not know | 1350 | 37.6 | 118 | 42.3 | 1229 | 37.2 | 3 | 25.0 |
| There is no benefit for PDD or NBI or IMAGE1S. | 1197 | 33.3 | 101 | 36.2 | 1092 | 33.1 | 4 | 33.3 |
| . |  |  |  |  |  |  |  |  |
| Agree | 366 | 10.2 | 16 | 5.7 | 348 | 10.5 | 2 | 16.7 |
| Disagree | 1022 | 28.4 | 77 | 27.6 | 942 | 28.5 | 3 | 25.0 |
| I do not know | 1010 | 28.1 | 85 | 30.5 | 922 | 27.9 | 3 | 25.0 |
| Multiple bladder tumors. | 1110 | 30.9 | 93 | 33.3 | 1013 | 30.7 | 4 | 33.3 |
| . |  |  |  |  |  |  |  |  |
| Agree | 1751 | 48.7 | 124 | 44.4 | 1623 | 49.1 | 4 | 33.3 |
| Disagree | 267 | 7.4 | 25 | 9.0 | 241 | 7.3 | 1 | 8.3 |
| I do not know | 467 | 13.0 | 37 | 13.3 | 427 | 12.9 | 3 | 25.0 |
| Larger bladder tumor including smaller satellite lesions. | 1144 | 31.8 | 94 | 33.7 | 1046 | 31.7 | 4 | 33.3 |
| . |  |  |  |  |  |  |  |  |
| Agree | 1692 | 47.1 | 125 | 44.8 | 1563 | 47.3 | 4 | 33.3 |
| Disagree | 310 | 8.6 | 21 | 7.5 | 288 | 8.7 | 1 | 8.3 |
| I do not know | 449 | 12.5 | 39 | 14.0 | 407 | 12.3 | 3 | 25.0 |
| In patients with pTa tumors to reduce recurrence rate. | 1143 | 31.8 | 94 | 33.7 | 1045 | 31.6 | 4 | 33.3 |
| . |  |  |  |  |  |  |  |  |
| Agree | 1457 | 40.5 | 101 | 36.2 | 1353 | 41.0 | 3 | 25.0 |
| Disagree | 456 | 12.7 | 34 | 12.2 | 421 | 12.7 | 1 | 8.3 |
| I do not know | 539 | 15.0 | 50 | 17.9 | 485 | 14.7 | 4 | 33.3 |
| In patients with pT1 tumors to reduce recurrence rate. | 1133 | 31.5 | 92 | 33.0 | 1037 | 31.4 | 4 | 33.3 |
| . |  |  |  |  |  |  |  |  |
| Agree | 1655 | 46.0 | 119 | 42.7 | 1533 | 46.4 | 3 | 25.0 |
| Disagree | 291 | 8.1 | 23 | 8.2 | 267 | 8.1 | 1 | 8.3 |
| I do not know | 516 | 14.4 | 45 | 16.1 | 467 | 14.1 | 4 | 33.3 |
| In patients with CIS to better diagnose and treat CIS. | 1088 | 30.3 | 91 | 32.6 | 993 | 30.1 | 4 | 33.3 |
| . |  |  |  |  |  |  |  |  |
| Agree | 2063 | 57.4 | 158 | 56.6 | 1900 | 57.5 | 5 | 41.7 |
| Disagree | 86 | 2.4 | 3 | 1.1 | 83 | 2.5 | . | . |
| I do not know | 358 | 10.0 | 27 | 9.7 | 328 | 9.9 | 3 | 25.0 |
| Which guidelines do you predominantly follow for the diagnosis and treatment of NMIBC? (Please select the one that best applies) | 1049 | 29.2 | 88 | 31.5 | 957 | 29.0 | 4 | 33.3 |
| . |  |  |  |  |  |  |  |  |
| The national guidelines prepared by my own society. | 358 | 10.0 | 23 | 8.2 | 334 | 10.1 | 1 | 8.3 |
| The national guidelines modified from the AUA, the EAU, the NCCN or similar guidelines. | 790 | 22.0 | 57 | 20.4 | 731 | 22.1 | 2 | 16.7 |
| The AUA guidelines | 154 | 4.3 | 11 | 3.9 | 142 | 4.3 | 1 | 8.3 |
| The EAU guidelines | 1017 | 28.3 | 81 | 29.0 | 932 | 28.2 | 4 | 33.3 |
| The NCCN guidelines | 123 | 3.4 | 13 | 4.7 | 110 | 3.3 | . | . |
| The ICUD consensus reports | 4 | 0.1 | 1 | 0.4 | 3 | 0.1 | . | . |
| I do not follow specific guidelines. | 100 | 2.8 | 5 | 1.8 | 95 | 2.9 | . | . |

**Appendix Table-A2:** Gender-specific comparisons related to TURBT including the use of enhanced imaging in the Residents/Fellows cohort.

|  | Female | | Male | | Chi-square P-Value |
| --- | --- | --- | --- | --- | --- |
|  | N | Row % | N | Row % |  |
| How many TURBT for bladder cancer (primary or secondary) do you perform per month? (q7_TURBT_frequency) | 9 | 9.5 | 25 | 5.0 |  |
| None |  |  |  |  | 0.0015 |
| 1-2 | 27 | 28.4 | 159 | 31.7 |  |
| 3-5 | 28 | 29.5 | 167 | 33.3 |  |
| 6-10 | 18 | 18.9 | 72 | 14.3 |  |
| >10 | 13 | 13.7 | 79 | 15.7 |  |
| I usually take biopsies only from abnormal looking urothelium. (q0013_0001) | 67 | 70.5 | 349 | 69.5 |  |
| Agree |  |  |  |  | 0.57 |
| Disagree | 28 | 29.5 | 153 | 30.5 |  |
| I take mapping biopsies in >50% of cases with White Light from normal looking urothelium. (q0013_0002) | 35 | 36.8 | 197 | 39.2 |  |
| Agree |  |  |  |  | 0.79 |
| Disagree | 60 | 63.2 | 305 | 60.8 |  |
| I only take mapping biopsies from normal looking urothelium with White Light if cytology or urinary marker test is positive. (q0013_0003) | 58 | 61.1 | 354 | 70.5 |  |
| Agree |  |  |  |  | 0.091 |
| Disagree | 37 | 38.9 | 148 | 29.5 |  |
| I almost never take bladder biopsies. (q0013_0004) | 16 | 16.8 | 56 | 11.2 |  |
| Agree |  |  |  |  | 0.17 |
| Disagree | 79 | 83.2 | 446 | 88.8 |  |
| There is no real benefit. (q0018_0001) | 8 | 8.4 | 82 | 16.3 |  |
| Agree |  |  |  |  | 0.33 |
| Disagree | 60 | 63.2 | 315 | 62.7 |  |
| I do not know | 27 | 28.4 | 105 | 20.9 |  |
| To identify satellite lesions for a lower recurrence rate. (q0018_0002) | 68 | 71.6 | 395 | 78.7 |  |
| Agree |  |  |  |  | 0.48 |
| Disagree | 7 | 7.4 | 30 | 6.0 |  |
| I do not know | 20 | 21.1 | 77 | 15.3 |  |
| To perform more complete resections in the outer tumor margin. (q0018_0003) | 62 | 65.3 | 373 | 74.3 |  |
| Agree |  |  |  |  | 0.38 |
| Disagree | 14 | 14.7 | 58 | 11.6 |  |
| I do not know | 19 | 20.0 | 71 | 14.1 |  |
| To perform more complete resection on the tumor floor. (q0018_0004) | 46 | 48.4 | 274 | 54.6 |  |
| Agree |  |  |  |  | 0.32 |
| Disagree | 24 | 25.3 | 134 | 26.7 |  |
| I do not know | 25 | 26.3 | 94 | 18.7 |  |
| There is a benefit when using PDD. (q0019_0001) | 43 | 45.3 | 226 | 45.0 |  |
| Agree |  |  |  |  | 0.72 |
| Disagree | 11 | 11.6 | 56 | 11.2 |  |
| I do not know | 41 | 43.2 | 220 | 43.8 |  |
| There is a benefit when using NBI. (q0019_0002) | 42 | 44.2 | 264 | 52.6 |  |
| Agree |  |  |  |  | 0.091 |
| Disagree | 8 | 8.4 | 50 | 10.0 |  |
| I do not know | 45 | 47.4 | 188 | 37.5 |  |
| There is a benefit when using IMAGE1S. (q0019_0003) | 20 | 21.1 | 177 | 35.3 |  |
| Agree |  |  |  |  | 0.046 |
| Disagree | 8 | 8.4 | 42 | 8.4 |  |
| I do not know | 67 | 70.5 | 283 | 56.4 |  |
| There is no benefit for PDD or NBI or IMAGE1S. (q0019_0004) | 9 | 9.5 | 76 | 15.1 |  |
| Agree |  |  |  |  | 0.17 |
| Disagree | 38 | 40.0 | 219 | 43.6 |  |
| I do not know | 48 | 50.5 | 207 | 41.2 |  |
| Multiple bladder tumors. (q0020_0001) | 66 | 69.5 | 357 | 71.1 |  |
| Agree |  |  |  |  | 0.60 |
| Disagree | 12 | 12.6 | 54 | 10.8 |  |
| I do not know | 17 | 17.9 | 91 | 18.1 |  |
| Larger bladder tumor including smaller satellite lesions. (q0020_0002) | 65 | 68.4 | 364 | 72.5 |  |
| Agree |  |  |  |  | 0.43 |
| Disagree | 10 | 10.5 | 57 | 11.4 |  |
| I do not know | 20 | 21.1 | 81 | 16.1 |  |
| In patients with pTa tumors to reduce recurrence rate. (q0020_0003) | 48 | 50.5 | 281 | 56.0 |  |
| Agree |  |  |  |  | 0.82 |
| Disagree | 24 | 25.3 | 113 | 22.5 |  |
| I do not know | 23 | 24.2 | 108 | 21.5 |  |
| In patients with pT1 tumors to reduce recurrence rate. (q0020_0004) | 59 | 62.1 | 334 | 66.5 |  |
| Agree |  |  |  |  | 0.75 |
| Disagree | 16 | 16.8 | 65 | 12.9 |  |
| I do not know | 20 | 21.1 | 103 | 20.5 |  |
| In patients with CIS to better diagnose and treat CIS. (q0020_0005) | 83 | 87.4 | 424 | 84.5 |  |
| Agree |  |  |  |  | 0.41 |
| Disagree | . | . | 17 | 3.4 |  |
| I do not know | 12 | 12.6 | 61 | 12.2 |  |
| Which guidelines do you predominantly follow for the diagnosis and treatment of NMIBC? (Please select the one that best applies) (q0022) | 15 | 15.8 | 69 | 13.7 |  |
| The national guidelines prepared by my own society. |  |  |  |  | 0.33 |
| The national guidelines modified from the AUA, the EAU, the NCCN or similar guidelines. | 27 | 28.4 | 144 | 28.7 |  |
| The AUA guidelines | 5 | 5.3 | 27 | 5.4 |  |
| The EAU guidelines | 39 | 41.1 | 219 | 43.6 |  |
| The NCCN guidelines | 4 | 4.2 | 20 | 4.0 |  |
| I do not follow specific guidelines. | 5 | 5.3 | 23 | 4.6 |  |
